# Effect of age and cancer on peripheral immune cell subsets and their PD-1 and PD-L1 expression

**DOI:** 10.1186/2051-1426-3-S2-P253

**Published:** 2015-11-04

**Authors:** Italia Grenga, Lauren Lepone, Renee Donahue, Jacob Richards, Simon Metenou, Christopher R Heery, Ravi Madan, James L Gulley, Jeffrey Schlom

**Affiliations:** 1 Laboratory of Tumor Immunology and Biology, Center for Cancer Research, National Cancer Institute, National Institutes of Health, Bethesda, MD, USA; 2 Genitourinary Malignancies Branch, Center for Cancer Research, National Cancer Institute, National Institutes of Health, Bethesda, MD, USA; 3 Genitourinary Malignancies Branch, National Cancer Institute, National Institutes of Health, Bethesda, MD, USA

## Purpose

Immunotherapies aiming to interfere with the immune checkpoint molecule PD-1 (programmed death-1) and its ligand PD-L1 are currently being investigated in several clinical trials to treat cancer patients. Little is known about the effect of age or cancer on peripheral immune cell subsets or on their expression of PD-1 and PD-L1. The aim of this study was to assess the differences in immune cell subsets, focusing on PD-1 and PD-L1 expression, between young and old healthy donors, as well as between age-matched healthy donors and cancer patients.

## Methods

A total of 123 peripheral immune cell subsets were analyzed by flow-cytometry using 30 unique markers. The subsets included 9 standard subsets (CD4 and CD8 T cells, regulatory T cells (Treg), B cells, conventional dendritic cells (cDC), plasmacytoid DC (pDC), natural killer cells (NK), natural killer T cells (NKT), and myeloid derived suppressor cell (MDSC)), and 114 subsets relating to maturation and function. PD-1 and PD-L1 surface expression was analyzed in all relevant subsets. The frequency of immune cells were compared between young (under age 40, n=11) and old (over age 40, n=15) apparently healthy individuals to assess the effect of age. To investigate the effect of cancer, the older healthy individuals were also compared to age matched patients with several types of advanced cancer (over age 40, n=30).

## Results

Compared to young healthy individuals, older subjects had several notable differences, including lower levels of total CD8 T cells and naïve CD8 T cells, and higher levels of total NK cells and PD-1 positive CD4 T cells. Relative to age-matched healthy individuals, advanced cancer patients had noteworthy differences in several immune cell subsets, such as higher levels of total CD8 T cells and PD-L1 positive MDSC, and lower levels of total B cells and PD-L1 positive B cells.

## Conclusions

The frequencies of several peripheral immune cell subsets, including some that express PD-1 or PD-L1, are affected by both age and cancer.

